# The Influence of Nanopore Dimensions on the Electrochemical Properties of Nanopore Arrays Studied by Impedance Spectroscopy

**DOI:** 10.3390/s141121316

**Published:** 2014-11-11

**Authors:** Krishna Kant, Craig Priest, Joe G. Shapter, Dusan Losic

**Affiliations:** 1 School of Chemical and Physical Sciences, Flinders University, Bedford Park, Adelaide, SA 5042, Australia; E-Mails: Krishna.Kant@adelaide.edu.au (K.K.); joe.shapter@flinders.edu.au (J.G.S.); 2 School of Chemical Engineering, the University of Adelaide, Adelaide, SA 5005, Australia; 3 Ian Wark Research Institute, University of South Australia, Mawson Lakes, Adelaide, SA 5095, Australia; E-Mail: Craig.priest@unisa.edu.au

**Keywords:** anodization, porous alumina, Electrochemical Impedance Spectroscopy (EIS), nanopore arrays

## Abstract

The understanding of the electrochemical properties of nanopores is the key factor for better understanding their performance and applications for nanopore-based sensing devices. In this study, the influence of pore dimensions of nanoporous alumina (NPA) membranes prepared by an anodization process and their electrochemical properties as a sensing platform using impedance spectroscopy was explored. NPA with four different pore diameters (25 nm, 45 nm and 65 nm) and lengths (5 μm to 20 μm) was used and their electrochemical properties were explored using different concentration of electrolyte solution (NaCl) ranging from 1 to 100 μM. Our results show that the impedance and resistance of nanopores are influenced by the concentration and ion species of electrolytes, while the capacitance is independent of them. It was found that nanopore diameters also have a significant influence on impedance due to changes in the thickness of the double layer inside the pores.

## 1. Introduction

The unique properties of metal oxide nanopores and their similarity with biological nanopore structures have attracted considerable research attention and seen the use of nanopores in many applications [1,2]. The integration of biomolecules into nanopores and nanochannels has been recognized as a new approach for development of simple, inexpensive and highly sensitive biomolecule detection [3–5]. The sensitivity, low cost, simplicity, and easy miniaturization of electrochemical detection is particularly suited to the development of highly sensitive nanopore-based biosensors [6]. The sensitivity of these nanopore-based devices is highly influenced by molecular transport of analyte molecules to reach binding receptors inside the nanopores where the dimensions of the nanopore structures (pore diameters and lengths) are critical parameters. Hence, a better understanding of electrical and electrochemical properties of the nanopores is essential for development and optimization of these devices. Thus characterizations using electrochemical methods are important to understand how devices based on nano-confined pore structures function [7,8] but surprisingly there have been few electrochemical studies on nanopore arrays and membranes [9].

Electrochemical impedance spectroscopy (EIS) is often used to characterize a wide variety of electrochemical phenomena related to the solid state, porous materials, synthetic and biological membranes, and liquid electrolytes [10]. Moreover, it provides valuable information on the functional and structural characteristics of membrane systems. The electrochemical impedance study in the ion-exchange membrane system provided a good means of investigating the electrochemical characteristics of fouling phenomena (*i.e.*, deposition and accumulation of foulants on anion-exchange membranes) [11]. EIS is a powerful method for analyzing the complex electrical resistance of a system and possible changes of bulk properties and, therefore, it has been widely used for characterization of charge transport across nanopores in membranes and membrane/solution interfaces [10]. The applications of impedance sensors are not limited to gas and humidity sensors, *etc.*, but these impedance sensors are also used in biological sensor which have very sophisticated interdigitated electrode structures [12,13].

The nanoporous alumina (NPAs) prepared by an electrochemical anodization process have been very attractive for the development of nanopore biosensing devices, due to their uniform pore size, high aspect ratio, high surface area and straightforward and inexpensive fabrication. It has been demonstrated that the electron transfer resistance will decrease and the conductance increase as a result of an ion transfer inside the pores of NPA [14,15]. Previous studies demonstrated that the transport of ions or molecules passing through nanopore nanochannels is affected by steric and electrostatic effects [16–18]. The electrostatic effect is a long-range effect caused by the interaction between surface charges and the transporting ions [13,19]. This transport is shown to be dependent on pore size and number of pores and can be changed by the surface charge or ionic strength of the bulk solution, which can also change their transport inside the nanopores [7,20]. These studies clearly demonstrate the need for a detailed investigation on the influence of nanopore dimensions on their electrochemical properties and biosensing performance [21,22].

Herein the aims of this work were to use impedance spectroscopy to explore the effect of nanopore diameters and lengths on the electrochemical properties of NPAs, which are related to their application in sensing and molecular separation. NPAs with different diameter (25 nm, 45 nm, 65 nm) and pore lengths (4.5 μm, 8 μm, 13.5 μm, 18 μm) were used as a nanopore array platform and sodium chloride (NaCl) with different concentrations was used as the electrolyte for the measurements.

## 2. Experimental Section

**Figure 1.**
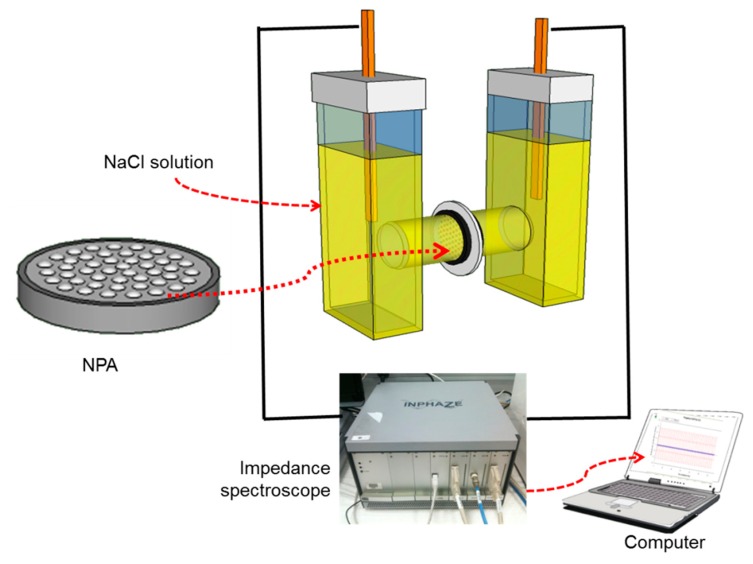
A schematic of experimental setup. The fabricated nano porous alumina (NPA) is sandwiched between two cells filled with electrolyte for two electrode EIS measurements of NPA.

The scheme of the proposed impedance devices and the experimental setup is shown in Figure 1. The NPA membranes were sandwiched between two different cell chambers and impedance measurements were performed by EIS.

### 2.1. Preparation of NPA

NPA membranes were prepared by a two-step anodization process using 0.3 M oxalic acid as electrolyte at 0 °C as described elsewhere [14]. The first anodized porous layer was prepared at a voltage of 30–60 V for 2–4 h, and then removed using an oxide removal solution (0.2 M chromium trioxide and 0.4 M phosphoric acid) for 1–2 h at 60 °C. The second anodization was carried out in the range of 30 V to 70 V to achieve different pore diameters. Mild anodization at 30 V was used to get pore diameters of 25 ± 5 nm and hard anodization at 70 V for large pore diameter of 65 ± 5 nm was used. The length of NPA is controlled by the time duration of anodization. For mild anodization at 30 V it took 180 min to achieve a pore length about 4–5 μm. However for hard anodization at 70 V it took only 20 min to grow 4–5 μm long pores [23]. To prepare NPAs with different thicknesses or lengths (5–18 μm) of small diameter pores the anodization time was varied from 180 min to 720 min at 30 V. Once the anodization was complete, the remaining aluminum was removed from the backside of the nanopore array by using CuCl_2_/HCl to reveal a clear and clean barrier layer of the nanopore array. The removal of barrier layer from the pore structures was performed by using 10% phosphoric acid [24].

### 2.2. Photolithography on NPA

The fabricated NPA was patterned on the back side (barrier layer side) with a photoresist using photolithography to prepare a 20 × 20 μm^2^ area with the opened nanopore array. The photoresist (AZ1518, AZ Electronic Materials, Somerville, NJ, USA) was spin-coated (1500 rpm) over the bottom side of the NPA (barrier layer) and followed by baking of the sample at 100 °C for 50 s. A photo-mask (chrome-on-glass mask, Bandwidth Foundry, Eveleigh, Sydney, NSW, Australia) with a 20 μm^2^ diameter transparent region was used to mask the sample with exposure of 330 mJ/m^2^. The exposed photoresist was then removed by solvent. In this way an opening of a 20 × 20 μm^2^ square on the NPA from bottom side was obtained.

### 2.3. Electrochemical Measurements

The transport characteristics of the prepared NPA membrane are determined by the behaviour of the nanopores, which forms the basis of our analysis. It was assumed that the solutions just inside and outside the pore are in equilibrium at the respective pore ends. If diffusion layers exist at the membrane-solution interfaces, as in our case, the solutions just inside the pore must be in equilibrium with the solutions at the membrane-diffusion layer interfaces. Electrochemical measurements were carried out using an EIS spectrometer (Inphaze, Pty. Ltd., Sydney, Australia) with a two-electrode system which is specifically designed for EIS characterization of NPA [9]. Two gold electrodes formed the working and counter electrodes. The nanoporous array was sandwiched between two cell chambers filled with the electrolyte each side. The working and counter electrodes were respectively placed at the end of two chambers (Figure 1). The Nyquist plot is one of the ways used to reveal the presence of various elements and processes as it provides a direct view of the manner in which the real component of the impedance (inverse of the conductance) varies with the imaginary component at each frequency. An equivalent circuit is used to fit the experimental data with the theoretical model. This model assumes that there is no impedance contribution made from the limited diffusion of the ions. In practice, such contribution does exist but mainly influences the impedance characteristics within a very low frequency range [25,26]. The resistance of the area of membrane, which is covered with photoresist, is so high that it does not appear in the equivalent circuit model. The pore resistance R_p_ can be calculated as:

(1) $$R_{p}=\frac{4L}{\pi \sigma d^{2}}$$

where R_p_ is pore resistance, L is length of the pore and d is the diameter of the pore and σ represents the specific conductivity of the electrolyte that fills the nanochannel. This equation indicates that an increase in the pore diameter will mean the pore resistance will reduce and the resistance will increase if we vary the length of the nanopores. The electrochemical impedances were acquired in the frequency range from 0.01 Hz to 1 MHz. In a nanopore system the conductance mostly depends on the pore diameter for high electrolyte concentration but also tends to have similar value for low concentrations as well. The conductance can be calculated using Equation (2):

(2) $$G_{0}=K_{b}{\left[\frac{4L}{\pi D^{2}}+\frac{1}{D}\right]}^{-1}$$

where K_b_ is the bulk conductivity, L length of nanopore and D is diameter of nanopore [27,28]. Studies already been done on the overlap of two electrical double layer (EDL) inside the nanopore [29]. The Debye length, the characteristic thickness of the EDL, is given by:

(3) $$\lambda_{D}={\left[\frac{ɛk_{B}T}{2e^{2}c_{s}}\right]}^{1/2}$$

where c_s_ is the salt concentration, ε is the dielectric permittivity of water, k_B_ is Boltzmann constant, T∼300 K is the absolute temperature, and e is the elementary charge [30,31]. In the case of very long nanopores both the ends and inner surface of the nanopore can be defined by Equation (2). The ends of nanopore produce system resistance and this effect means that each nanopore can be considered as one resistance in series. Hille and Hall [27,32] explained the pore electric conductance G_p_ is proportional to the bulk conductivity:

(4) $$G_{P}=K_{b}\left[\frac{\pi D^{2}}{4L}\right]$$

If the surface conduction of nanopore (Equation (4)) is combined with pore conductance, the total pore conductance can be calculated by the expression:

(5) $$G_{P}=\left[K_{b}\frac{\pi D^{2}}{4L}+K_{s}\frac{\pi D}{L}\right]$$

If the length L of the nanopore is large and the diameter is small the pore resistance (R_p_) dominates the total conductance of nanopore where K_s_ is the surface conductivity and K_b_ is the bulk conductivity [33]. The increase in the impedance across the pore is approximately equal to the applied potential meaning that the potential drops at the electrode/solution interfaces are small. The applied potential and generated resistance at interface may not be same on both sides of the membrane. R_p_ is small if the conductivity of the electrolyte is high and if the pore channel is very short, R_p_ becomes small.

## 3. Results and Discussion

### 3.1. SEM Characterization of NPA Arrays

**Figure 2.**
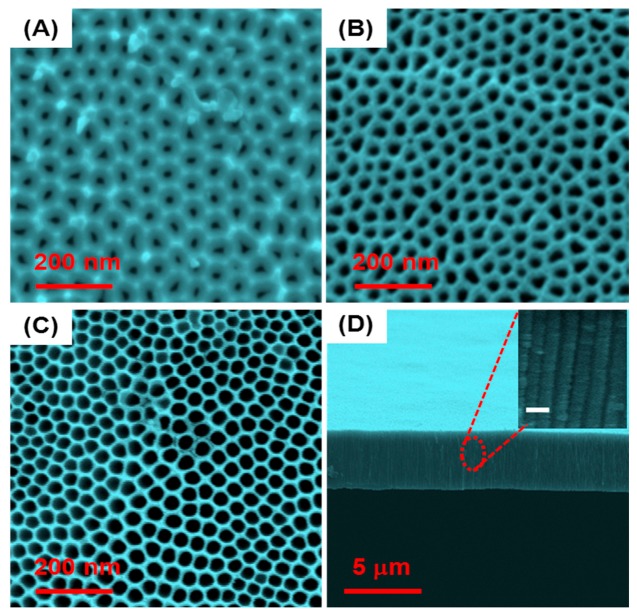
SEM images of the top surface of NPA used as nanopore sensing platform with different pore diameters (**A**) small pore around 25 ± 2 nm (**B**) medium size pore around 45 ± 2 nm (**C**) large pore around 65 ± 2 nm, prepared in 0.3 M oxalic acid electrolyte using different anodization voltages 30–70 V and times between 20 to 180 min to achieve the same pore length (**D**) Cross section SEM images of NPA with the inset figure (scale bar 100 nm) showing well-ordered and aligned pores through the full thickness of the membrane.

SEM images of the top surface of the prepared NPA sensing platform with different pore diameters are presented in Figure 2a–c. The images confirm the successful preparation of well-ordered nanopores with a diameter of 25 ± 2 nm, 45 ± 2 nm and 65 ± 2 nm, depending on the anodization conditions. To achieve three different pore diameters at constant pore length the anodization potential was tuned between 30 and 70 V with the anodization time; varied from 20 to 180 min. It is well known that pore diameter is determined by applied voltage during anodization and that the porosity (P) of the NPA can be estimated assuming an ideal hexagonal arrangement of the pores:

(6) $$P=\left(\frac{\pi }{2\sqrt{3}}\right){\left(\frac{D_{p}}{D_{\text{int}}}\right)}^{2}$$

where D_p_ and D_int_ are pore diameter and the inter-pore distance respectively [34]. A typical cross-sectional structure of the prepared NPA is shown in Figure 2d.

### 3.2. EIS Measurements on NPA Arrays: Influence of Ion Concentration

**Figure 3.**
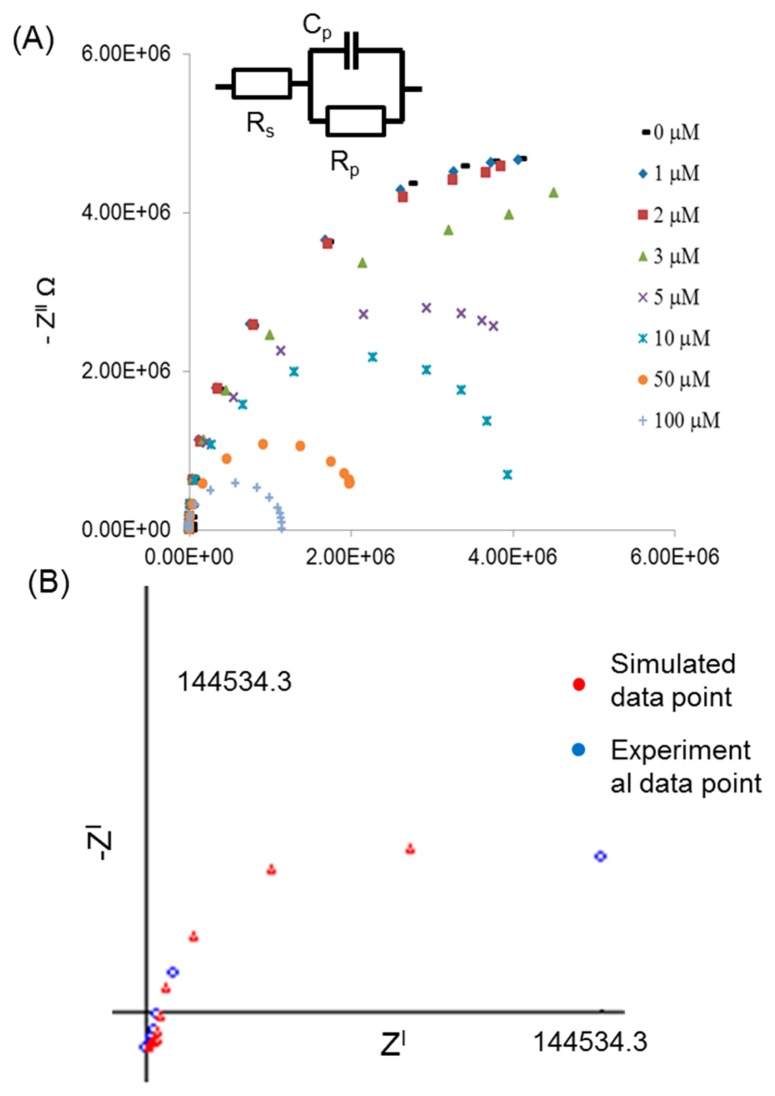
(**A**) Nyquist plots obtained for NPA at constant pore diameters (45 nm) of with various different concentrations of NaCl (**B**) experimental data is showing the agreement against the simulated data with use of equivalent circuit.

In order to characterize the performance of these NPA arrays and hence allow the optimization of the nanopore dimensions for sensing applications, a series of electrochemical impedance spectroscopy experiments were carried out [35,36]. In the first set of experiments, NPA arrays were characterized using NaCl electrolyte with concentrations ranging from 1 to 100 μM. The collected impedance (Z) data for the constant pore diameters using various concentration of NaCl are shown in Nyquist plots (Figure 3). The Nyquist plot consists of over lapping semicircles, each of which is associated with a single time constant element. The apex of the semicircle corresponds to the characteristic frequency of elements in the system. The decrease in the radius of the semicircle at high concentration is due to the diffusion of ions inside the nanopores and increase in the conductance of the nanopore array especially at the very high concentration. Surface conductivity, K_s_, changes due to high concentration of ions at the end of the nanopore while bulk conductivity, K_b_, has opposite impact on the system conductance and reduces the resistivity. The Nyquist plot provides a more immediate indication of the ion diffusion due to the concentration gradation. It reveals the effect of concentration on the conductivity of the NPA. The graphs show that Z^l^ (real) decreased with increasing electrolytic concentration inside pores of NPA.

To estimate the impedance of the NPA, we modeled the experimental system using the EvolCRT software developed at the Department of Chemistry, Wuhan University (Wuhan, China) [37]. Here, R_s_ is resistance of the electrolyte solution; R_p_ and C_p_ are the total contribution of resistance and capacitance of alumina membranes (Figure 3a). The experimental data was simulated against the equivalent circuit to find out the exact value for the pore resistance (R_p_). The R_p_ is a key parameter which is directly related to changes happening due to the diameter of nanopore. The wall thickness of NPA and available NaCl ions inside the nanopores generates an EDL on inner surface of nanopores which resulted in high impedance inside the nanopores. Therefore, R_P_ is much larger than R_S_. It is also found that both R_S_ and R_P_ decrease with increasing concentration indicating that the nanopore resistance is a function of the electrolyte concentration inside the nanopore, so the high concentration ratio induced by the trans-membrane potential increases the mobility of the charged ions inside the nanopore resulting in a relatively lower electrolyte resistance [38].

### 3.3. EIS Measurements on NPA Arrays: Influence of Nanopore Diameters and Length

**Figure 4.**
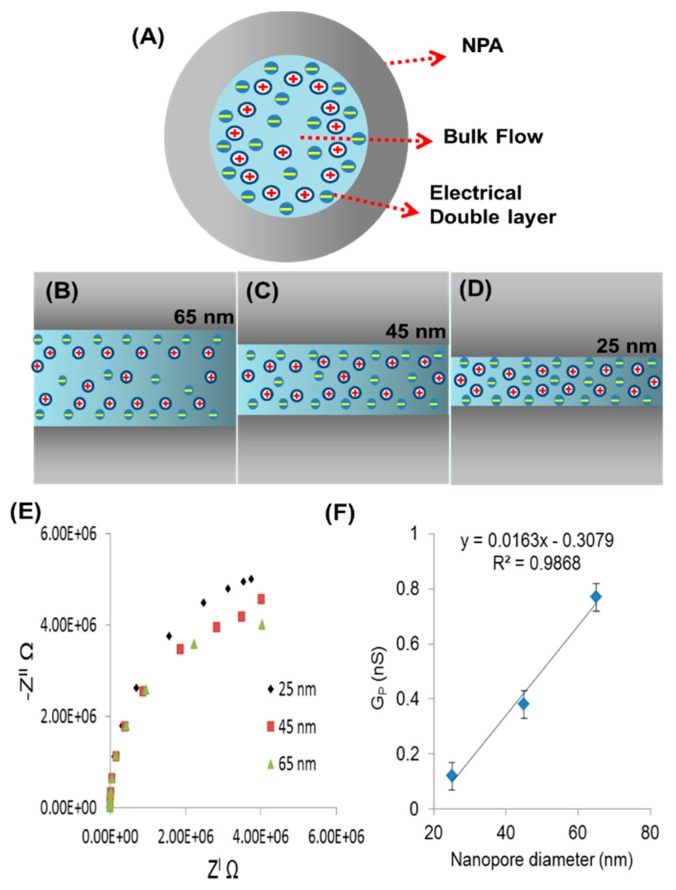
Schematic presentation for formation of electrical double layer and bulk flow of electrolyte (**A**) inside the nanopore (**B**) large pore diameters (65 nm) has space for bulk flow of electrolyte but as pore diameter decreases (**C**) and (**D**) the density of ions increases inside nanopores (**E**) Nyquist plot between the three different pore diameters 25 nm, 45 nm and 65 nm of NPA at constant length 4.5 μm and concentration (1 μM) of electrolyte NaCl shows the decrease in pore resistance R_p_ with increased pore diameter; (**F**) increase in electric conductance (G_p_) with respect to pore diameter.

NPAs of three different pore diameters (25, 45, and 65 nm) were characterized using NaCl electrolyte. The collected impedance (Z) data for the various pore diameters using constant concentration of 1 μM NaCl is shown in Nyquist plots (Figure 4e). The values for R_p_ obtained after simulation with equivalent circuit shown in Figure 3a. The graph showing the influence of pore diameter on R_p_. At the lowest concentration of electrolyte 1 μM, the pore resistance decreases from 5.61 × 10^6^ to 3.8 × 10^6^ Ω when the pore diameter is increased from 25 to 65 nm. For the large pore diameter, the impedance measurement is dominated by the bulk properties of the buffer solution (Figure 4a,b) and small changes in impedance due to effects at the walls of the nanopores become insignificant. The conductance of a nanopore (G_p_) is also affected by the increase in the diameter of nanopore (Equation (3)) as the diameter is increasing (25, 45 and 65 nm) conductance increases (0.12, 0.38 and 0.77 nS), respectively (Figure 4f). The effect of electrical double layer depends on the electrolyte concentration and increases with decrease in electrolyte concentration. In case of large nanopores (65 nm) there is enough space for the ions to diffuse inside the nanopore so the electric double layer is not affecting system resistance. However, in the case of the smaller pores (Figure 4d), the electrical double layer (at the same concentration of electrolyte) formed on each side of the alumina wall is very close to each other and starts to effect ion movement inside the nanopore causing high resistance in the system. The relative surface effects are greater and the signal is affected significantly, as reported in previous studies [39–41]. The conclusion from this experiment is that the NPA with smaller nanopore diameter (25 nm) is comparatively more sensitive (∼120 Ω/μM) than the larger pores (∼45 Ω/μM), which was found over the lower range (1 μM to 50 μM) of concentration.

**Figure 5.**
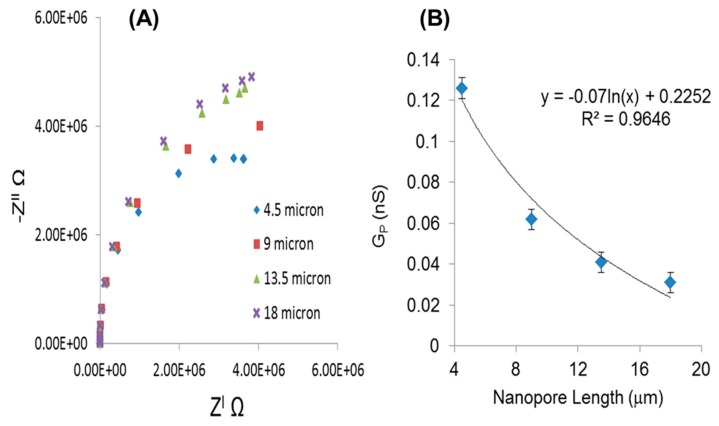
(**A**) At constant pore diameter (25 nm) and concentration (1 μM) four different pore lengths shows the increase in pore resistance R_p_ with increased pore length (**B**) change in electric conductance (G_p_) with respect to nanopore length.

Since we observed that the smaller pore diameter provides greater sensitivity and performance, we used the 25 nm diameter pores to study the influence of pore length on electrochemical signal. For that purpose we fabricated NPA biosensing platforms with different pore lengths (4.5, 9, 13.5 and 18 μm) and carried out a series of EIS measurements at different concentrations of NaCl. The Nyquist plots generated at a constant concentration of electrolyte (1 μM) are shown in Figure 5a. The graph shows the trend of increasing impedance over the range of frequencies with respect to increasing pore length. The calculated G_p_ decreases (0.126, 0.062, 0.041 and 0.031 nS) with increasing nanopore length respectively (Figure 5b). Further measurements were performed by simulating the experimental data with the proposed equivalent circuit to find value of R_p_ for various length of NPA. It was found that Rp changes from 3.2 × 10^6^ to 5.32 × 10^6^ Ω as the length of NPA increases from 4.5 to 18 μm. The graph clearly shows the increase in the pore resistance with increasing the pore length. The characteristic saturation in signal was observed at higher concentration of electrolyte [42]. It was found that increasing the pore length beyond 10–15 μm lead to higher system resistances, as ion path will be longer inside nanopore and density of ion will not be in equilibrium at both ends of the nanopore. Increasing nanopore length to more than 10 to 15 μm will not be useful for biosensing detection where biomolecules need to diffuse through the nanopores [43].

It was observed that pore resistance is a key factor to be considered in the optimization of NPA performance by EIS. In our case the pore resistance with respect to lower concentration (1–10 μM) has a high sensitivity for small change in concentration of electrolyte. This behavior of NPA arrays is also limited with the morphology of nanopore array. A small nanopore diameter (below 20 nm) is also been effected by the electrical double layer and has a low conductance [44,45]. The detection limit for electrolyte using this system was 1 μM. However we believe a detection limit can be improved by lowering nanopore diameters (<25 nm). Nanopores with a diameter below 20 nm were difficult to make using the anodization conditions employed in our experiments (oxalic acid anodization), but the use of other electrolytes (*i.e.*, sulphuric acid) will allow the production of systems with smaller pores. However, smaller nanopores may limit the diffusion of the ions and other molecules such as protein and enzymes. The best performance for the biosensing platform was observed with nanopore diameters of 20–40 nm and a length of 4–15 μm.

## 4. Conclusions

The EIS method was successfully applied to measure the impedance characteristics of NPA nanopore sensing platforms to explore the influence of the pore dimensions on their electrochemical properties. Our results confirmed that pore diameter and length have considerable influence on device performance where pore resistance is used as the sensing method. The results indicate that smaller pores provide increased sensitivity with optimal pore diameters in range of 20–40 nm. However, there is a limit for the pore size reduction down to sizes (<10 nm) comparable to the size of analyte molecules which will prevent diffusion molecules inside the pore. In terms of pore length, it was found that optimal lengths are in 5–15 μm range. Longer pores (>15 μm) are also not favorable for non-Faradic EIS detection as a result of higher nanopore impedance and long diffusion time of analyte molecules inside the nanopores. It is critical to optimize pore dimensions to achieve optimal performances of nanopore based electrochemical biosensing devices which have considerable potential toward the development simple and inexpensive sensing devices for broad biomedical applications.
